# TiO_2_ nanotubes wrapped with reduced graphene oxide as a high-performance anode material for lithium-ion batteries

**DOI:** 10.1038/srep36580

**Published:** 2016-11-03

**Authors:** Peng Zheng, Ting Liu, Ying Su, Lifeng Zhang, Shouwu Guo

**Affiliations:** 1 School of Materials Science and Engineering, Shaanxi University of Science and Technology, Xian 710021, Shaanxi, P. R. China; 2 Department of Electronic Engineering, School of Electronic Information and Electrical Engineering, Shanghai Jiao Tong University, Shanghai 200240, P. R. China

## Abstract

Through electrostatic interaction and high-temperature reduction methods, rGO was closely coated onto the surface of TiO_2_ nanotubes. Even at a high temperature of 700 °C, the nanotube morphology of TiO_2_ (anatase) was preserved because of the assistance of rGO, which provides a framework that prevents the tubes from breaking into particles and undergoing a phase transformation. The rGO/TiO_2_ nanotubes deliver a high capacity (263 mAh g^−1^ at the end of 100 cycles at 0.1 A g^−1^), excellent rate performance (151 mAh g^−1^ at 2 A g^−1^ and 102 mAh g^−1^ at 5 A g^−1^), and good cycle stability (206 mAh g^−1^ after 500 cycles at 0.5 A g^−1^). These characteristics arise from the GO/TiO_2_ nanotubes’ advanced structure. First, the closely coated rGO and Ti^3+^ in the tubes give rise to a high electro-conductivity of the nanotubes. Additionally, the Li^+^ ions can rapidly transfer into the electrode via the nanotubes’ empty inner diameter and short tube wall.

With increasing development of electric vehicles (EVs), hybrid electric vehicles (HEVs) and wind/solar energy, more stringent requirements are being placed on Li-ion batteries (LIBs) as the stationary energy storage devices for these techologies^1^,^2^,^3^. High capacity, outstanding rate capability and long cyclic stability are the main performance targets for LIB electrode materials^4^,^5^,^6^. Among the anode materials developed thus far, titanium dioxide (TiO_2_) has the advantage of a small volume change (less than 4%) and a relatively high working voltage (1.5 V vs Li^+^/Li), making it a promising electrode material for meeting the aforementioned challenges^7^,^8^,^9^. The small volume change of this material leads to good long-term cycling stability, and the high working voltage results in a small irreversible capacity and high safety by avoiding the formation of solid electrolyte interphase (SEI) layers.

Whereas anatase TiO_2_ has a high theoretical capacity of 335 mAh g^−1^, its poor electronic and ionic conductivities limit its practical capacity and rate performance^10^,^11^,^12^,^13^. Pore generation (meso/hierarchical pores and hollow structure)^9^,^14^,^15^, Ti^3+^ doping (via hydrogenation or Mg reduction)^16^,^17^, the use of special facets ((001), (101) or (010) facets)^12^,^18^ and carbon coupling are effective approaches for overcoming the aforementioned barriers because these approaches can provide a shorter ion diffusion length and a greater electronic conductivity. In particular, much outstanding work has been reported on the carbon-coupling (TiO_2_/C and TiO_2_/graphene) approach^19^,^20^,^21^,^22^. In recent years, various TiO_2_/C composite structures have been fabricated, including mesoporous TiO_2_ wrapped in carbon^8^, graphitic carbon coating of mesoporous TiO_2_ hollow spheres^9^ and nanostructured CNT@TiO_2_-C^23^. Because of its outstanding conductivity and good structure flexibility, the TiO_2_/graphene composite is a promising candidate composite material. Many impressive structures have been demonstrated. For instance, Zhao *et al.*^24^ demonstrated that anatase nanoparticles ultra-dispersed onto graphene exhibit a high specific capacity of 94 mAh g^−1^ at 59C. Zhang *et al.*^25^ showed that mesoporous anatase nanoparticles grown on graphene aerogels could deliver a capacity of 202 mAh g^−1^ at 0.59C. Song *et al.*^26^ showed that a sandwich-like porous anatase/reduced graphene oxide electrode exhibited a capacity of 206 mAh g^−1^ at 0.59C. Furthermore, the TiO_2_ mesocrystals/reduced graphene oxide synthesized by Wei *et al.*^20^ was reported to deliver 150 mAh g^−1^ at 20C after 1000 cycles. These studies suggest that the design and modulation of various morphologies of TiO_2_ and graphene can lead to high capacity and good rate performance, depending on their special structures, because of the flexibility of graphene and the rich morphology of TiO_2_.

In this report, we employ titanate nanotubes as a substrate and coat graphene oxide onto their surface via electrostatic interaction. After being annealed at 700 °C under a 5% H_2_/Ar mixed atmosphere, graphene oxide was reduced to graphene and was closely coated onto the surface of the TiO_2_ nanotubes, providing a framework and preventing the nanotubes from breaking into particles. Simultaneously, Ti^3+^ was generated in the TiO_2_ tube walls. We observed that when used as an LIB anode, this composite exhibits a high capacity of 263 mAh g^−1^ at 0.1 A g^−1^ (0.59C) in the voltage window from 1.0 to 3.0 V. Excellent rate performance (151 mAh g^−1^ at 2 A g^−1^ and 102 mAh g^−1^ at 5 A g^−1^) and good long-term cycling stability (500 cycles without decaying) were also obtained. These excellent performances are attributed to the unique structure of the composite material. First, the empty space inside the nanotubes and the thin tube walls are beneficial for the diffusion of Li^+^ ions. Second, the closely coated reduced graphene oxide frame can support faster electron transfer to a TiO_2_ electrode. Finally, the introduced Ti^3+^ can improve the conductivity of the TiO_2_ electrode.

## Results and Discussion

**Figure 1:**
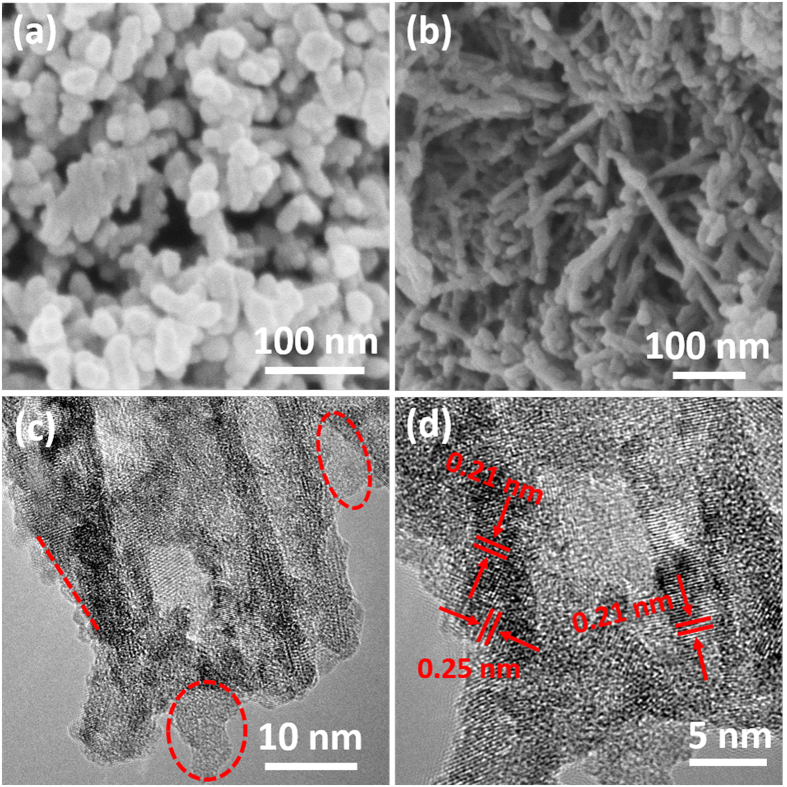
(**a**) FE-SEM image of titanate nanotubes calcined at 500 °C under an Ar atmosphere; (**b**) FE-SEM image of TiO_2_/rGO-7ArH, the inset is the corresponding EDX spectrum; (**c,d**) HRTEM images of TiO_2_/rGO-7ArH.

The titanate nanotube precursor was synthesized using the NaOH hydrothermal method^27^, which resulted in nanotubes with a diameter and length of approximately 18 and 220 nm (Fig. S1a and S1b), respectively. To wrap reduced graphene oxide (rGO) on the surface of the titanate nanotubes, electrostatic interactions^28^ and high-temperature reduction methods were used, as described in detail in the Experimental section. The products were labeled as rGO/TiO_2_-XY (where X represents the annealing temperature and Y represents the annealing atmosphere). The wrapped un-calcined sample is shown in Fig. S1c; many nanopores are observed in the aggregated titanate nanotubes. In the case of the sample annealed at 500 °C under Ar atmosphere, the unwrapped nanotubes were broken into nanoparticles (Fig. 1a). By contrast, the nanotube morphology was still preserved in the wrapped samples, even those annealed at 700 °C (Fig. S1d and 1e). The closely coated rGO layer on the nanotube plays a key role in this preservation of morphology, providing a frame for the tube and preventing the TiO_2_ from undergoing a phase transformation and breaking into nanoparticles. Additional Ti^3+^ would be introduced under a substantially stronger reducing atmosphere, which is favorable for improving the conductivity^29^. Therefore, the sample was also calcined under an H_2_/Ar mixture atmosphere at 700 °C. Figure 1b,c and d show the detailed morphologies of rGO/TiO_2_-7ArH. Numerous nanopores are observed among the loose aggregated nanotubes (Fig. S1f). As shown in the enlarged FE-SEM image in Fig. 1b, the nanotubes maintained their original size. As indicated in the EDX spectrum (inset of Fig. 1b), the nanotubes are composed of carbon, oxygen and titanium. The presence of rGO was revealed by HRTEM. In Fig. 1c, the region circled in red is the rGO. Additionally, the lattice fringes corresponding to the interlayer spacing of (200) and (002) of TiO_2_ (anatase) are also observed (Fig. 1d). As is well-known, higher annealing temperatures usually lead to better crystallinity. However, the poor degree of crystallinity observed in Fig. 1d is not consistent with its high synthesis temperature. We conjecture that many Ti^3+^ and oxygen vacancy defects are generated under the reduced (5% H_2_/Ar mixture) atmosphere.

**Figure 2:**
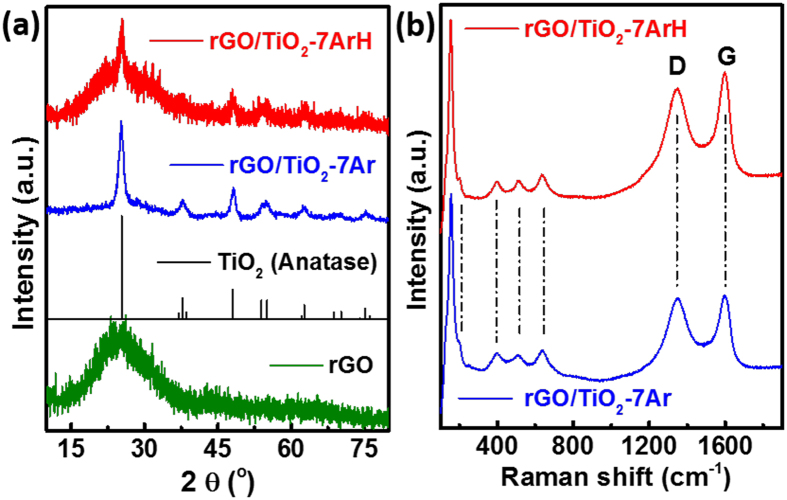
(**a**) XRD patterns of rGO, the standard TiO_2_ sample (JCPDS: 99-0008), rGO/TiO_2_-7Ar and rGO/TiO_2_-7ArH; (**b**) Raman spectra of rGO/TiO_2_-7Ar and rGO/TiO_2_-7ArH.

Figure 2a shows the XRD patterns of the synthesized samples; all peaks of the samples were indexed to TiO_2_ (anatase, JCPDS: 99-0008) and rGO. Because of the high crystallinity of rGO/TiO_2_-7Ar, the low-intensity, broad rGO peak at 2θ = 24.5° is obscured by a TiO_2_ peak. By contrast, in the case of rGO/TiO_2_-7ArH, because the presence of many Ti^3+^ defects lowers the TiO_2_ crystallinity, the hump rGO peak at 2θ = 24.5° is clearly observed.

To provide further evidence for the structure of the prepared samples, we collected their Raman spectra. As shown in Fig. 2b, we observed five characteristic Raman scattering peaks at 145 (Eg), 198 (Eg), 398 (B1g), 516 (A1g + B1g), and 638 (Eg) cm^−1^ corresponding to the peaks for tetragonal anatase TiO_2_ crystals^30^ and the typical D and G bands for rGO^31^. Because of the much stronger reducing atmosphere of the H_2_/Ar mixture compared to Ar, the intensity of the G band in the spectrum of rGO/TiO_2_-7ArH is greater than that in the spectrum of rGO/TiO_2_-7Ar. On the basis of these data, combined with the results of the XRD investigation, we concluded that the synthesized samples are composed of rGO and anatase TiO_2_. In the case of rGO/TiO_2_-7ArH, the rGO content was estimated as 7%, as revealed by the TG analysis (Fig. S2).

**Figure 3:**
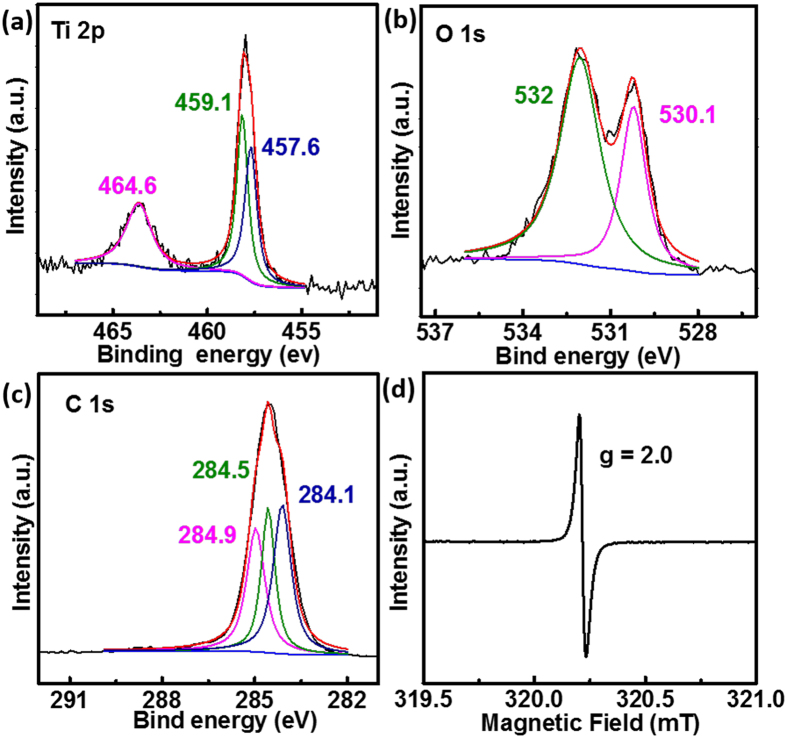
(**a**) Ti 2p, (**b**) O 1s, and (**c**) C 1s XPS spectra of rGO/TiO_2_-7ArH; (**d**) EPR spectrum of rGO/TiO_2_-7ArH collected at room temperature.

To confirm the presence of the Ti^3+^ defects, we conducted high-resolution XPS. Two peaks of Ti 2p_1/2_ (464.6 eV) and Ti 2p_3/2_ (458.2 eV) are observed in Fig. 3a; after curve fitting, the Ti 2p_3/2_ was deconvoluted into two peaks centered at 459.1 eV and 457.6 eV. The 457.6 eV peak is related to Ti^3^+ 32,33. In the high-resolution O 1s spectrum (Fig. 3b), two peaks centered at 532 eV and 530.1 eV are observed; these peaks correspond to oxygen vacancy defects and to crystal lattice oxygen species O^2−^, respectively^32^,^34^. Thus, the presence of Ti^3 +^ and oxygen vacancy defects in rGO/TiO_2_-7ArH is verified. The C 1s spectrum was deconvoluted into three peaks, including the sp^2^-C bonds of graphene (284.1eV), sp^3^-hybridized carbons (C-C/ C-H, 284.5 eV) and alcohol, epoxy, and ether groups (C-O, 284.9 eV). No Ti-C or Ti-O-C bond was observed^35^,^36^. Room-temperature EPR was also used to confirm the presence of Ti^3+^; as shown in Fig. 3d, a resonance signal at g = 2.0, corresponding to Ti^3+^, was observed in the EPR spectrum of rGO/TiO_2_-7ArH. The lack of a Ti^3+^ signal for common TiO_2_^37^,^38^ implies that many Ti^3+^ ions were introduced into rGO/TiO_2_-7ArH.

**Figure 4:**
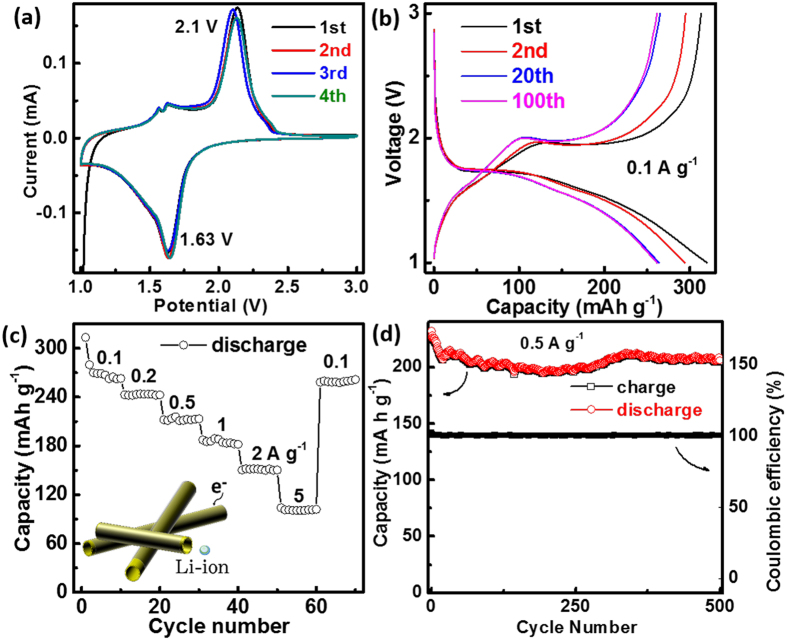
Electrochemical properties of rGO/TiO_2_-7ArH composites: (**a**) CV measurements at a scan rate of 0.5 mV s^−1^, (**b**) discharge–charge profiles at 0.1 A g^−1^, (**c**) rate performances, and (**d**) cycling performances at 0.5 A g^−1^.

The electrochemical performance of the produced samples as anodes for LIBs was tested using Li metal as the counter electrode assembled into CR2032 cells. Cyclic voltammetry (CV) revealed the Li^+^-ions (de-)intercalation mechanism into/out of the electrode. After four consecutive scans at a rate of 0.1 mV s^−1^ for rGO/TiO_2_-7ArH, the resulting curves were observed to overlap well with each other, indicating good cycle stability of the electrode (Fig. 4a). The pure capacity of rGO was 70 mAh g^−1^ between approximately 1.0 and 3.0 V (Fig. S3). Furthermore, a pair of cathodic/anodic peaks (1.63/2.1 V) associated with Li insertion/extraction was also observed. The overall reaction mechanism can be expressed using Eq. (1):


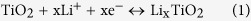


The maximum lithium insertion coefficient was approximately 0.5 (Li0.5TiO2)^39^; on this basis, 1C = 167.5 mA g^−1^. The charge-discharge profiles at 0.1 A g^−1^ (0.59C) are presented in Fig. 4b; these profiles show that the system delivers a discharge capacity of 320, 294, 263 and 262 mAh g^−1^ for the 1st, 2nd, 20th and 100th cycles, respectively. In the charge-discharge profiles, the voltage plateaus coincide well with the CV peaks, interpreted as corresponding to the tetragonal anatase transforming into orthorhombic Li_0.5_TiO_2_ upon Li insertion^40^,^41^.

Figure 4c displays the excellent rate performance of rGO/TiO_2_-7ArH. At current densities of 0.1, 0.2, 0.5, 1, 2 and 5 A g^−1^, the discharge capacities are 262, 243, 212, 183, 151 and 102 mAh g^−1^, respectively. When the current density was decreased to 0.1 A g^−1^, the capacity recovered to 262 mAh g^−1^. Such excellent rate performance arises from the special structure shown in the inset of Fig. 4c. First, the close coating of rGO and the intrinsic Ti^3+^ of the TiO_2_ electrode favor good electron transport^42^, thus solving the problem of poor electro-conductivity of TiO_2_.

Thus, the inner void of the tube is beneficial for Li^+^-ion diffusion, whereas the thin walls of the nanotubes provide a short migration distance, ensuring fast Li^+^-ion diffusion ability. The thin wall also favors long cycle stability; the accumulated stress is smaller for a thin wall, thus preserving the structural integrity and enabling a high reversible capacity. After 500 cycles, the capacity is as high as that after the 20th cycle (205 mAh g^−1^ at 0.5 A g^−1^) and the Coulombic efficiency approaches 100% during all of the cycles (Fig. 4d).

**Figure 5:**
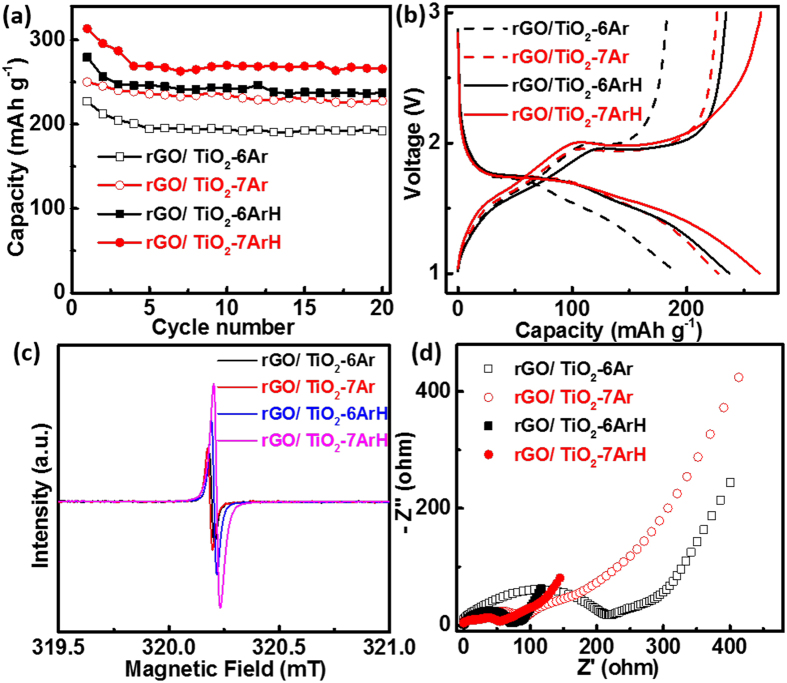
(**a**) Cycling performance, (**b**) discharge–charge curves of the 20th cycle at 100 mA g^−1^ in the range from 1.0 to 3.0 V; (**c**) EPR spectra; (**d**) Nyquist plots for the EIS data of rGO/TiO_2_-6Ar, rGO/TiO_2_-7Ar, rGO/TiO_2_-6ArH and rGO/TiO_2_-7ArH.

The effects of the annealing temperature and atmosphere on the LIB performance were also investigated. Figure 5a shows the capacities of the samples synthesized under various conditions, with the corresponding discharge–charge curves at the 20th cycle displayed in Fig. 5b. rGO/TiO_2_-6Ar, rGO/TiO_2_-7Ar, rGO/TiO_2_-6ArH and rGO/TiO_2_-7ArH exhibit capacities of 192, 227, 237 and 265 mAh g^−1^, respectively, at the end of the 20th cycle at 0.1 A g^−1^. The performance of the samples annealed under a H_2_/Ar mixture atmosphere is better than that of the samples annealed under Ar at the corresponding temperatures, and high temperatures favor enhanced capacity. In addition to the role of rGO, the generated Ti^3+^ also strongly affects various performance characteristics, improving the conductivity, as previously reported^29^,^41^. With higher temperatures and stronger reducing atmospheres, more Ti^3+^ is generated, as verified by EPR (Fig. 5c). Among the EPR spectra, that of rGO/TiO_2_-7ArH exhibits the highest intensity, whereas that of rGO/TiO_2_-6Ar exhibits the lowest intensity. The improved conductivity is also reflected in the electrochemical impedance spectroscopy (EIS) results. As shown in Fig. 5d, in the high-frequency region, a single semicircle is identified as the charge-transfer resistance (R_ct_) in the Nyquist plots for the rGO/TiO_2_ electrodes. A smaller diameter of the semicircle indicates a lower R_ct_ of the electrode and better conductivity. rGO/TiO_2_-7ArH exhibited the lowest R_ct_ value among the investigated samples.

## Conclusions

In summary, we have prepared rGO/TiO_2_ nanotube composites through electrostatic interaction and high-temperature reduction methods. The closely coated rGO offers a framework that prevents the TiO_2_ nanotube from breaking up and undergoing a phase transformation under high temperatures. Under an H_2_/Ar mixture atmosphere at 700 °C, a large amount of Ti^3+^ is generated. Because of the advantageous properties of rGO and Ti^3+^, the electro-conductibility of TiO_2_ is improved and Li^+^ ions can rapidly diffuse into the electrode via the inner space and the thin walls of the nanotubes. Thus, rGO/TiO_2_-7ArH exhibits excellent performance as an anode for LIBs. The high capacity (263 mAh g^−1^ at the end of 100th cycle at 0.59C), excellent rate performance (151 mAh g^−1^ at 2 A g^−1^ and 102 mAh g^−1^ at 5 A g^−1^) and good cycle stability (no capacity decay after 500 cycles) make the rGO/TiO_2_ nanotube composite a promising anode material for high-performance LIBs.

## Experimental

### Preparation of titanate nanotubes

The titanate nanotubes were synthesized using the hydrothermal method^27^. Typically, 0.3 g of TiO_2_ (P25) was transferred into a Teflon reactor and suspended in 40 mL of 10 M NaOH. The reactor was sealed and placed into an autoclave for 12 h at 130 °C. After the reaction, the sample was washed with deionized water and 0.1 M HCl. All samples were dried at 80 °C for 12 h.

### Preparation of graphene-wrapped TiO_2_ nanotubes

TiO_2_ nanotubes wrapped with reduced graphene oxide were synthesized through modification of their electrostatic interaction^28^. Typically, 0.3 g of titanate nanotubes was dispersed in 100 mL of ethanol by stirring for 6 h. Then, 2 mL of 3-aminopropyl-trimethoxysilane (APTMS) was added, and the resulting mixture was refluxed at 95 °C for 4 h. APTMS-treated titanate nanotubes were rinsed with sufficient ethanol to wash away the remaining APTMS. Then, 3 mL of the negatively charged GO suspension (5 mg mL^−1^) was added to the positively charged amine-functionalized titanate nanotube dispersion under vigorous stirring. After being stirred for 1 h, the mixture was centrifuged, washed with deionized water and dried at 60 °C for 12 h. Finally, the product was calcined at different temperatures under various atmospheres for 2 h to remove organic components and obtain crystalline graphene-TiO_2_ nanotubes. In the case of calcination under an Ar atmosphere at 600 and 700 °C, the corresponding samples were labeled as rGO/TiO_2_-6Ar and rGO/TiO_2_-7Ar, respectively. The samples calcined under a 5% H_2_/Ar mixture atmosphere at 600 and 700 °C were labeled as rGO/TiO_2_-6ArH and rGO/TiO_2_-7ArH, respectively.

### Materials Characterization

X-ray diffraction (XRD) analysis was performed using a Rigaku-Dmax 2200 diffractometer equipped with a Cu-Kα radiation source. Field-emission scanning electron microscopy (FE-SEM) was performed using a field-emission Rigaku S4800 electron microscope equipped with an energy-dispersive X-ray (EDX) spectrometer (Thermo Scientific NSS). High-resolution transmission electron microscopy (HRTEM) was performed on an FEI Tecnai F20 instrument. X-ray photoelectron spectroscopy (XPS) analysis was carried out using an ULVAC-PHI5000 X-ray photoelectron spectrometer. Thermogravimetric analysis (TG) (NETZSCH STA 409PC, Germany) was performed at a heating rate of 10 °C min^−1^ under flowing air. Raman spectra were collected using a micro-Raman spectrometer (Invia) with a laser operating at a wavelength of 532 nm. Room-temperature electron paramagnetic resonance (EPR) was carried out using a JES FA200 spectrometer.

### Electrochemical Measurements

Electrochemical measurements were performed using CR2032 type coin cells with Li metal as the counter/reference electrode, a rGO/TiO_2_ film as the working electrode, and 1 M LiPF_6_ (Aldrich 99.99%) dissolved in an EC/DEC solution with a 1:1 volume ratio. Cu foil was used for electrical connection to the rGO/TiO_2_ film. The working electrodes of control samples were prepared by mixing 80 wt% active material, 10 wt% conducting carbon black, and 10 wt% polyvinylidene fluoride binder in *N*-methyl-2-pyrrolidone. The loading density of the electrode on the current collector was 1.24 mg cm^−2^. The cells were assembled in an argon-filled glove box. Galvanostatic charge–discharge cycles were conducted on a Newaresles battery cycler at various current densities at potentials between 1.0 and 3 V vs. Li^+^/Li at room temperature. Cyclic voltammetry (CV) and electrochemical impedance spectroscopy were carried out at room temperature using an electrochemical workstation (CHI 660E).

## Additional Information

**How to cite this article**: Zheng, P. *et al.* TiO_2_ nanotubes wrapped with reduced graphene oxide as a high-performance anode material for lithium-ion batteries. *Sci. Rep.* **6**, 36580; doi: 10.1038/srep36580 (2016).

**Publisher’s note:** Springer Nature remains neutral with regard to jurisdictional claims in published maps and institutional affiliations.

## Supplementary Material

Supplementary Information
