# Supplementary material for: TiO2 nanotubes wrapped with reduced graphene oxide as a high-performance anode material for lithium-ion batteries
Source: Sci Rep. 2016 Nov 3;6:36580. doi: 10.1038/srep36580 (PMC5093559; doi:10.1038/srep36580)
Supplement: Supplementary Information [file srep36580-s1.pdf]

TiO_2 nanotubes wrapped with reduced graphene oxide as a high-performance anode material for lithium-ion batteries

Peng Zheng, *[a] Ting Liu,[a] Ying Su,[a] Lifeng Zhang,[a] and Shouwu Guo, *[a,b]

a School of Materials Science and Engineering, Shaanxi University of Science and Technology, Xian 710021, Shaanxi, P. R. China.
b Department of Electronic Engineering, School of Electronic Information and Electrical Engineering, Shanghai Jiao Tong University, Shanghai 200240, P. R. China

zhengpeng@sust.edu.cn; swguo@sjtu.edu.cn

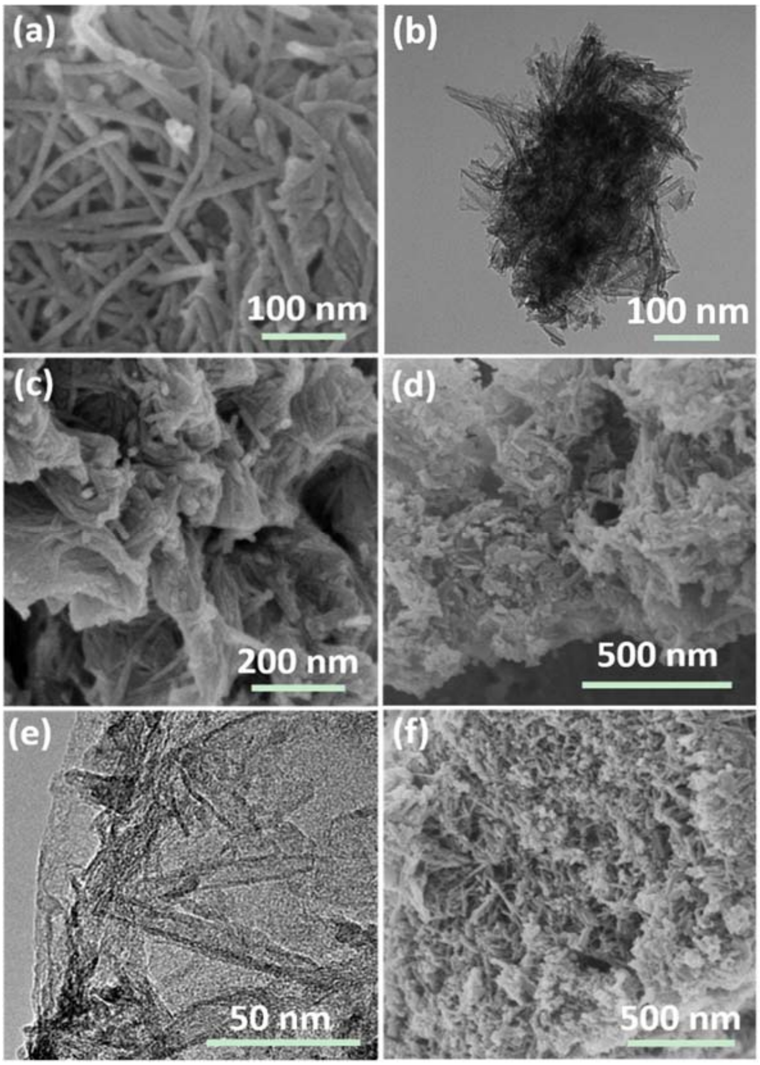

Figure S1. (a) FE-SEM and (b) TEM images of titanate nanotubes; (c, d) FE-SEM and (e) HRTEM images of TiO_2/rGO-7Ar; (f) FE-SEM image TiO_2/rGO-7ArH.

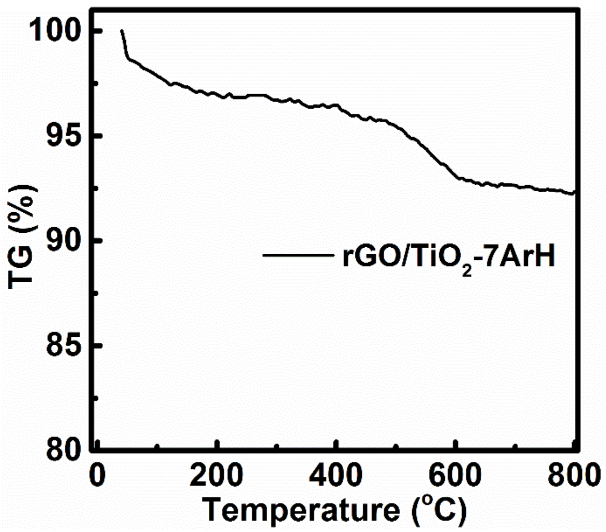

Figure S2. Thermogravimetric analysis of rGO/TiO_2-7Ar under air atmosphere. Heating rate: 10 °C min^{-1}.

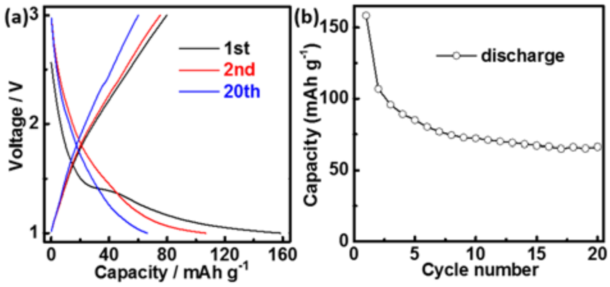

Figure S3. (a) Discharge–charge curves, (b)Cycling performance of rGO at 100 mA g^{-1} in the range of 1.0– 3.0 V;
